# Case Report: A patient with metastatic fumarate hydratase-deficient renal cell carcinoma associated with leiomyomatosis: real-world clinical insights on systemic therapy and liver-directed SBRT

**DOI:** 10.3389/fonc.2026.1729830

**Published:** 2026-02-18

**Authors:** Huili James Chong, Alastair Murray, Kwang Jeat Chong

**Affiliations:** 1 Department of Oncology, Mahkota Medical Centre, Melaka, Malaysia; 2 Department of Anatomical Pathology, Canterbury Health Laboratories, Christchurch, New Zealand

**Keywords:** bevacizumab and erlotinib, fumarate hydratase-deficient renal cell carcinoma, hereditary leiomyomatosis and renal cell carcinoma, stereotactic body radiation therapy, uterine leiomyomatosis

## Abstract

Fumarate hydratase-deficient renal cell carcinoma is a rare type of renal cell carcinoma often associated with hereditary leiomyomatosis and renal cell carcinoma syndrome. These tumors tend to exhibit an aggressive behavior and metastasize at an early stage. We describe the case of a 41-year-old woman of Chinese ethnicity who presented with progressive left flank pain and macroscopic hematuria. Computed tomography (CT) scan of the abdomen showed a large renal mass occupying the entire left kidney, left renal vein and inferior vena cava (IVC) tumor thrombus extension, left renal hilar lymphadenopathy, and indeterminate iliac bony and multiple large uterine fibroids. The patient underwent radical nephrectomy, caval thrombectomy and IVC reconstruction, total abdominal hysterectomy, and bilateral salpingo-oophorectomies. Histopathological examination revealed metastatic fumarate hydratase-deficient renal cell carcinoma associated with uterine leiomyomatosis with R1 resection margin at IVC. Owing to the lack of uniformly agreed guidelines for the management of this tumor, close surgical surveillance was advised. The patient subsequently developed postoperative liver metastases and sought self-funded medical care abroad. She subsequently received bevacizumab and erlotinib and achieved a favorable response. However, the patient experienced renal impairment with proteinuria following treatment, and the next cycle of systemic therapy was delayed. During this pause, we proceeded with stereotactic body radiation therapy (SBRT) to the remaining solitary liver metastasis. This case illustrates the practical challenges faced in treating fumarate hydratase-deficient renal cell carcinoma, including the lack of established systemic treatment guidelines and management of treatment-related adverse events. It highlights the value of integrating radiotherapy during interruptions in systemic therapy and the importance of multidisciplinary collaboration in this rare tumor.

## 1 Introduction

Fumarate hydratase-deficient renal cell carcinoma (FH-dRCC) is a new molecularly defined renal carcinoma, according to the World Health Organization’s fifth edition of the Classification of Tumors Editorial Board (1). This term is preferred over the previous term, “hereditary leiomyomatosis and renal cell carcinoma (HLRCC) syndrome,” as not all FH-RCCs are hereditary, with some arising sporadically due to somatic mutation (1).

HLRCC is an inherited autosomal dominant condition caused by alterations in the FH gene (2). Patients with HLRCC are at risk of developing uterine leiomyomata, cutaneous leiomyomata, or RCC (2). The reported prevalence of HLRCC is 1 per 200,000 (3). Furthermore, a 2019 cohort study in the United Kingdom reported that approximately 12.4% of patients with HLRCC develop kidney tumors (3). HLRCC-associated RCC is known to exhibit aggressive behavior, although the reasons remain unclear, and has the propensity to metastasize early despite a small tumor size with up to 75% of patients diagnosed with distant metastasis at presentation (4, 5). The most common sites of metastasis at presentation are retroperitoneal lymph nodes (81%), lungs (50%), and thoracic lymph nodes (38%) followed by liver, bone, and adrenal involvement (31%) (6). Overall survival varies with reports of a median survival of 21.9 months in a cohort receiving mixed treatment regimens including mTOR and VEGF combinations, VEGF monotherapy, checkpoint inhibitor therapy, and mTOR monotherapy (6). The combination of bevacizumab and erlotinib achieved a median overall survival of 45 months in a phase II trial (7). Because of the rarity of this malignancy and current evidence limited by single-arm trial designs and small sample sizes, the established treatment regimen remains elusive.

We report the case of a 41-year-old Chinese ethnicity woman with no family history of cancer who presented with aggressive malignancy and elected to have a hysterectomy at the same time for symptomatic fibroids. She underwent a complex, multispecialty surgery involving gynecology, urology, hepatobiliary, and vascular teams; however, she developed multiple liver metastases. The patient responded well to bevacizumab and erlotinib therapy and received stereotactic body radiation therapy (SBRT) to target the remaining solitary liver and indeterminate bony lesion. This report highlights the challenges she faced and the value of integrating radiotherapy during systemic therapy pauses.

## 2 Case description

A 41-year-old woman of Chinese descent presented with 1 year of progressive left flank pain, which the patient initially attributed to musculoskeletal back pain. Weeks of increased urinary frequency and macroscopic hematuria prompted the patient to seek medical attention. At presentation, she had an ECOG performance status of 0. The patient had no significant medical or smoking history and did not consume alcohol. In her family history, her mother had a history of fibroids. There was no family history of malignancy.

The urine dipstick test was negative for glucose, ketones, blood, protein, nitrite, and leucocytes. Complete blood count showed mild normocytic anemia, a hemoglobin level of 109 g/L (reference range, 115–155 g/L), and a mean corpuscular volume of 93 fL (reference range, 80–99 fL). White cell count was reduced, 3.3 ×109/L (reference range, 4.0–11.0 ×109/L); lymphopenia, 0.6 ×109/L (reference range, 1.0–4.0 ×109/L). Neutrophil count was 2.0 ×109/L (reference range, 1.9–7.5 ×109/L) and platelet count was 217 ×109/L (reference range, 150–400 ×109/L), which were within normal limits. Renal function was largely preserved, with a serum creatinine level of 80 μmol/L (reference range, 45–90 μmol/L) and an estimated glomerular filtration rate of 79 mL/min/1.73 m² (reference range, 80–120 mL/min/1.73 m²). Liver function tests were within normal limits: serum albumin, 37 g/L (reference range, 32–38 g/L); alanine aminotransferase (ALT), 18 U/L (reference range, 0–30 U/L); alkaline phosphatase (ALP), 70 U/L (reference range, 30–150 U/L); and total bilirubin, 12 μmol/L (reference range, 2–20 µmol/L).

The urology team reviewed the patient, who requested a computed tomography (CT) scan of the abdomen and pelvis. CT revealed a large expansile heterogeneous renal mass occupying the entire left kidney (85 × 73 × 106 mm); extensive left renal vein and inferior vena cava (IVC) tumor thrombus extension; left renal hilar lymphadenopathy; an indeterminate sclerotic bony lesion within the left iliac bone indeterminate; multiple large uterine fibroids; and left-sided abdominal and pelvic varices likely related to the renal vein tumor thrombus.

The case was discussed at a urology–radiology meeting; open radical nephrectomy and caval thrombectomy with the input of vascular surgery, general surgery, and gynecology specialties were planned. The patient underwent a 6-h radical nephrectomy, caval thrombectomy, total abdominal hysterectomy, and oophorectomy. Intraoperative assessment confirmed tumor invasion of suprarenal IVC. Thus, en bloc resection of the involved IVC segment was performed, followed by reconstruction using a tabularized bovine pericardial graft. The estimated intraoperative blood loss was 2,000 mL, and the patient was admitted to the intensive care unit for close monitoring.

**Figure 1:**
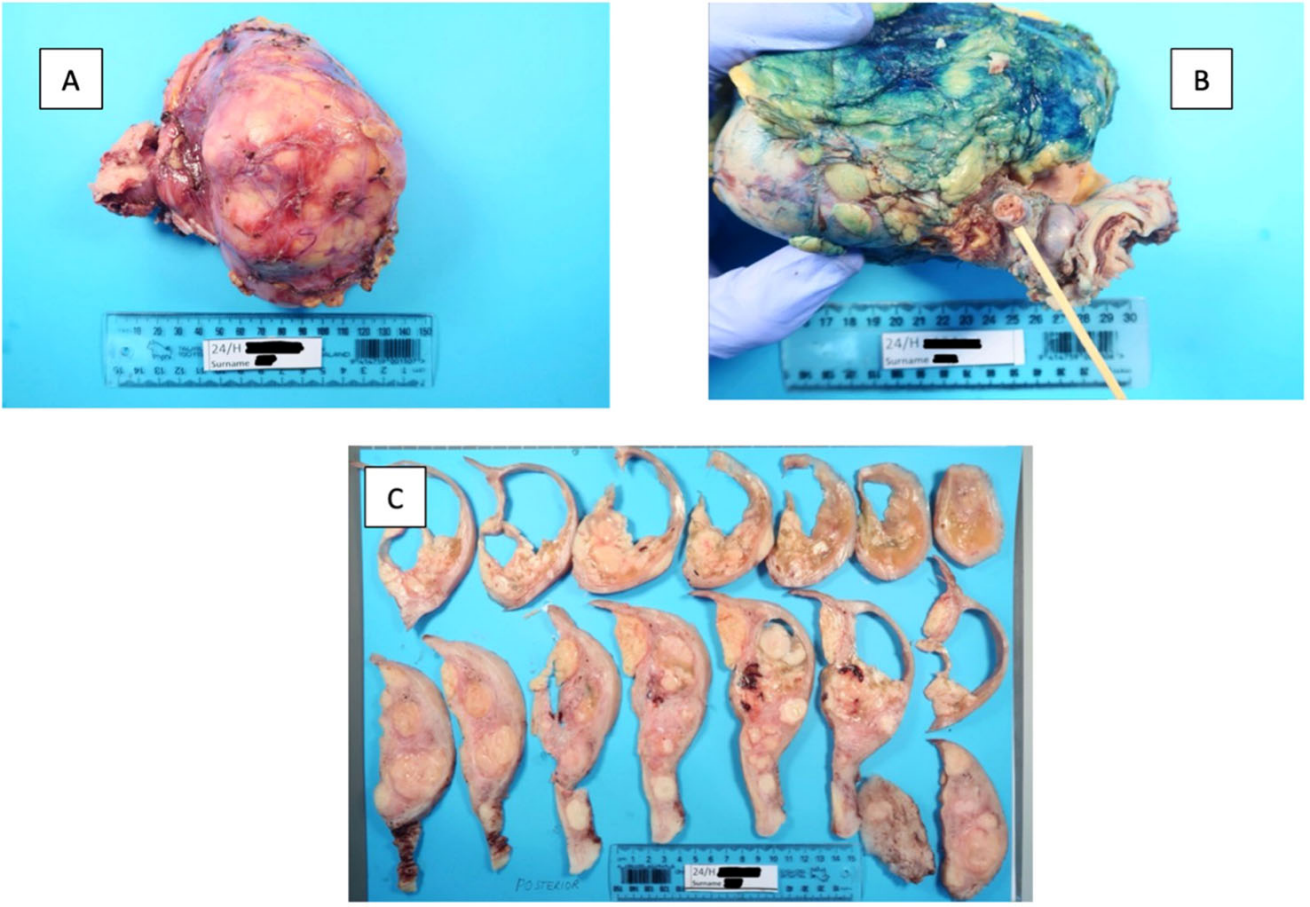
Gross images of **(A)** renal tumor, **(B)** renal vein and branch involvement, and **(C)** hysterectomy; numerous fibroids were found to be metastasis from the renal cancer.

**Figure 2:**
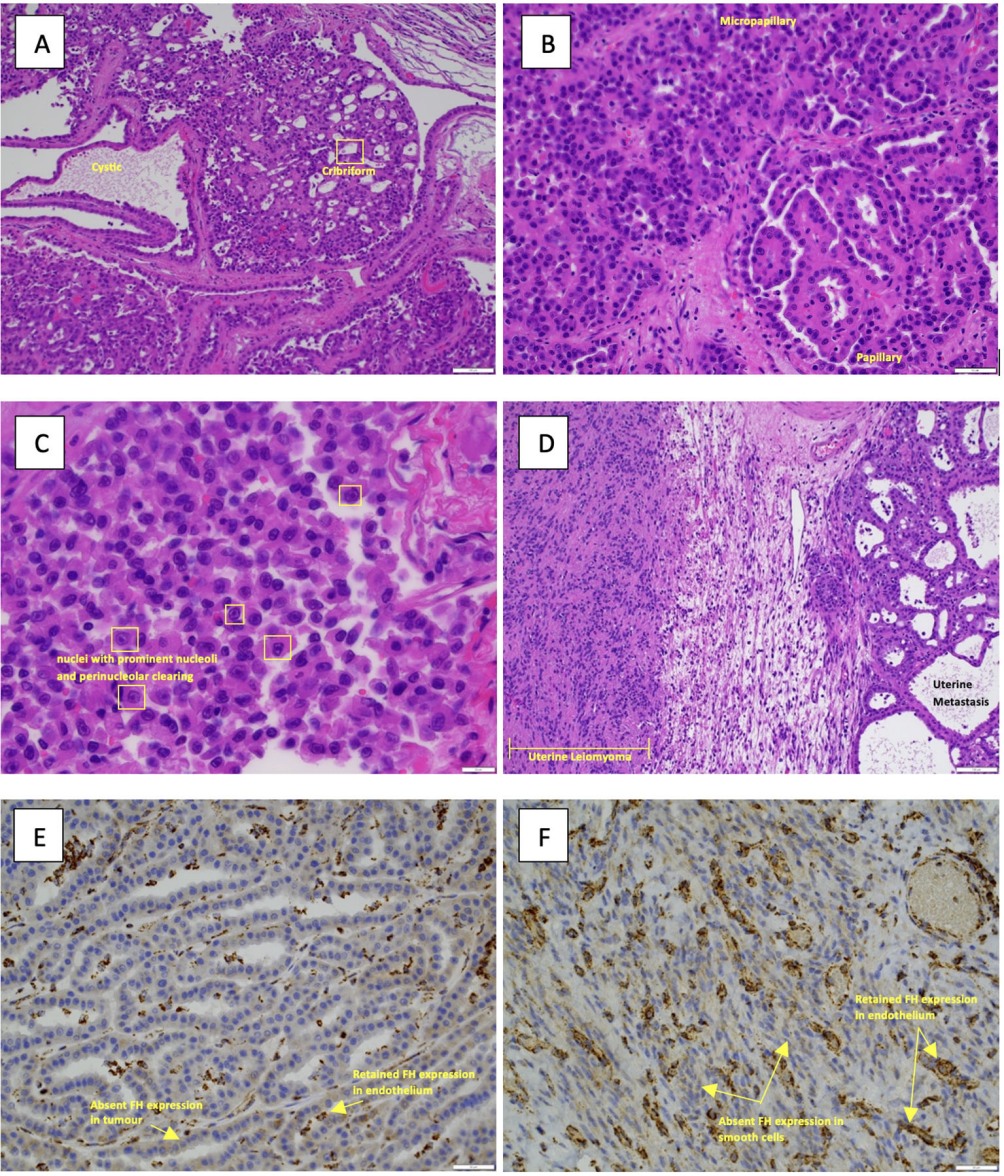
**(A)** Hematoxylin and eosin (H&E) image of the primary renal tumor illustrating the various growth patterns present, a feature of FH-deficient RCC with cystic, cribriform areas (10× magnification). **(B)** H&E image of the primary renal tumor illustrating papillary and micropapillary areas (10× magnification). **(C)** H&E image, at high power (40× magnification), nuclei with prominent nucleoli and perinucleolar clearing seen. **(D)** Microscopy image of the uterine metastasis with an adjacent leiomyoma (10× magnification). **(E)** The immunohistochemical stain is fumarate dehydrogenase (40× magnification), demonstrating loss of expression of FH in the tumor cells but retained expression in the endothelial cells (acting as an internal control). **(F)** Fumarate hydratase (40× magnification) of the leiomyoma showing loss of expression in the smooth muscle cells and retained expression in the blood vessels.

The pathology report provided the following details (**Figure 1**, **2**). 1) The renal tumor (130x75x70mm) showed areas of varied architecture with cysts, cribriform, sieve-like areas, and papillary growth. The cells had an abundant eosinophilic cytoplasm, characteristic eosinophilic nucleoli with a surrounding clear halo. 2) FH-deficient leiomyomas and a metastasis of the FH-deficient RCC were identified in the uterus, and this is strongly suggestive of HLRCC syndrome. 3) The left kidney showed FH-RCC, pT3bN0M1, R1 resection margin at the IVC site, stage IV. Tumor necrosis, sarcomatoid and rhabdoid differentiation were absent. Four lymph nodes were examined, all of which were negative for metastatic disease. Ancillary Findings: The tumor cells stain positive for cytokeratin cocktail (CKCO), vimentin, and PAX8. They are absent for fumarate-hydratase (i.e. FH-deficient), GATA3, oestrogen receptors, and cytokeratin 7.

**Figure 3:**
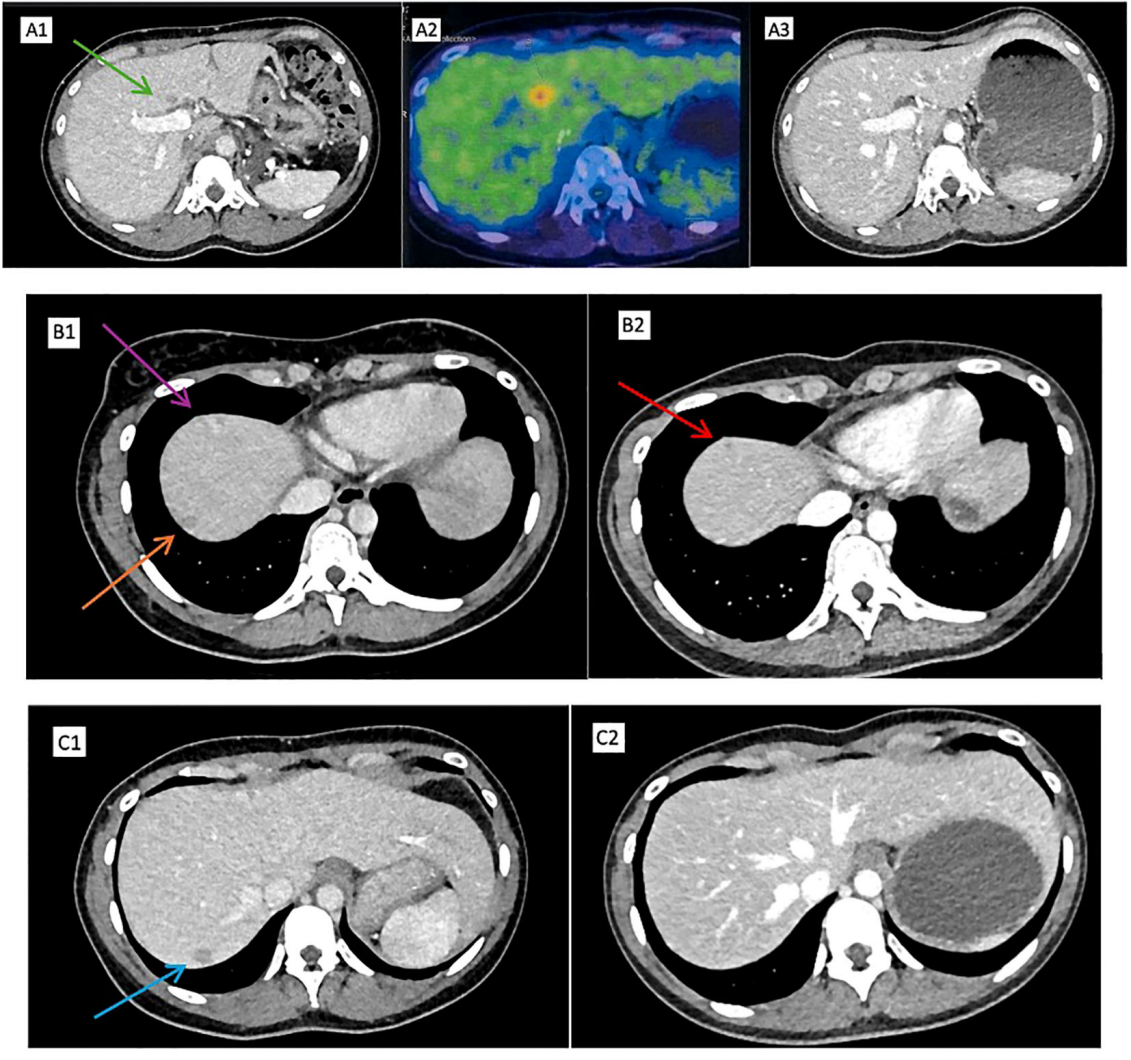
Positron emission tomography (PET)–computed tomography (CT): **(A1)** Hypodense lesion in segment 4 (green arrow). **(A2)** FDG-avid irregular hypodense liver lesion at segment 4 (SUVMax 5.1) after cycle 1. **(A3)** Complete resolution of hypodense liver lesion in segment 4 after cycle 4. **(B1)** Hypodense liver lesions in segment 8 (purple arrow) and segment 7 (orange arrow). **(B2)** Smaller hypodense liver lesion in segment 8 after cycle 4 (red arrow). **(C1)** Hypodense liver lesion in segment 7. **(C2)** Complete resolution of hypodense liver lesion in segment 7 after cycle 4.

The patient was reviewed in an outpatient follow-up clinic 8 weeks later; the urologist advised in the absence of evidence to support routine postoperative systemic therapy, and close surgical surveillance was recommended. The patient was referred to a clinical geneticist for genetic consultation and possible surveillance strategies for family members. The patient was also referred to a private medical oncologist in New Zealand, who similarly emphasized the lack of evidence for postoperative systemic therapy and advised close surgical surveillance. A follow-up CT scan of the abdomen and pelvis 14 weeks after surgery showed several hypodense round liver lesions with liver metastases of up to 15 mm in the S4, S7, and S8 liver segments (**Figure 3**).

Because of uncertainty over treatment eligibility and the known aggressive behavior of this tumor, the patient decided to travel briefly to Malaysia for self-funded treatment. Germline testing was discussed with the patient and the patient elected to wait for a genetics clinic appointment in New Zealand. During this short stay in Malaysia, she was started on bevacizumab (400 mg; weight, 49 kg) and erlotinib (100 mg once a day). At the time of treatment initiation, the patient met two criteria and thus was classified as intermediate-risk disease by IMDC. IMDC risk stratification was used for prognostic context, recognizing its limitations in FH-deficient RCC and in patients treated with bevacizumab and erlotinib (8). Positron emission tomography (PET)–CT 3 weeks later showed partial response (RECIST 1.1 criteria) to treatment as an FDG-avid solitary liver metastasis measuring 11 × 13 mm at segment 4, and a suspected left iliac bone lesion was observed (**Figure 3**). However, as her renal impairment deteriorated, eGFR of 37 mL/min/1.73 m² from a baseline of 55 mL/min/1.73 m² post-nephrectomy, with a proteinuria level of 0.74 g/24 h (24-h protein excretion), the second cycle of bevacizumab and erlotinib treatment was delayed. With uncertainty over the duration of interruption of systemic therapy and recovery of renal function, we decided to proceed with SBRT to the solitary liver metastasis (40 Gy/3 #) and bone metastasis to the ilium (24 Gy/3 #). The patient was also referred to a nephrologist who recommended the discontinuation of an angiotensin-converting enzyme (ACE) inhibitor (Quinapril 5 mg once a day) and the initiation of an angiotensin receptor blocker (ARB; 50 mg of Losartan once a day) for renal protection and sodium bicarbonate 650 mg twice a day for 4 weeks to address concurrent metabolic acidosis. The patient returned to New Zealand and subsequently had funding approved to resume bevacizumab and erlotinib 6 weeks after administration of cycle 1.

The patient underwent a CT scan of the abdomen and pelvis after four cycles of systemic therapy, which showed partial response (RECIST 1.1 criteria), the complete resolution of segment 4 and 7 liver metastasis, and residual segment 8 lesions reduced to 3.5 and 8 mm with no new lesions (**Figure 3**). Another CT scan of the abdomen and pelvis after four further cycles showed stable appearances (RECIST 1.1 criteria) with no new metastatic disease.

## 3 Discussion

As highlighted in the largest case series of 185 individuals from 69 families, FH-deficient RCC can develop in 12.4% of patients with HLRCC (3). This tumor shows characteristically a variety of growth patterns, with the majority of cases showing a papillary pattern, followed by solid, tubulocystic, cribriform, and cystic tumors (9). The hallmark feature of this lesion is the microscopic appearance of a large eosinophilic nucleolus surrounded by a clear halo (10). The *FH* gene is located at chromosome 1q43, and the median age of onset is typically reported to be in the fourth decade of life, as in our patients, with the youngest patient being 7 years old in Japan (9, 11). Our patient had similar varying histological growth patterns but all with scattered cells with characteristics eosinophilic nucleoli and a surrounding halo.

Regarding clinical manifestations of HLRCC, recent studies have suggested that cutaneous leiomyomatosis occurs in 46%–48% of patients with HLRCC (3, 12), which is considerably lower than earlier estimates of 76%–100% (13, 14). This discrepancy may be attributed to ascertainment bias. Uterine leiomyomas are observed in approximately 77% of affected individuals, often presenting in women in their early thirties and frequently necessitating surgical intervention and oral contraceptives for symptomatic relief (14–16). In our case, multiple large uterine fibroids were observed on abdominal CT and dermatological examination revealed no evidence of cutaneous leiomyomata.

The diagnostic criteria for HLRCC syndrome proposed by Schmidt et al. include one major criterion, three minor criteria, and a definitive diagnosis of a positive germline FH mutation (17). The diagnosis of HLRCC in our patient has not yet been confirmed. Histopathological examination showed loss of FH staining in the uterine leiomyoma and renal tumor. Loss of fumarate hydratase (FH) leads to fumarate accumulation, which causes downstream protein succination forming 2-succincysteine (2SC) adducts, a metabolic hallmark of FH-deficient RCC. Recent studies have suggested that 2SC IHC is considered a more sensitive marker (9, 18). In our case, 2SC IHC was not available in New Zealand and the nearest laboratory with this stain was in Australia. As the tumor was shown unequivocally to be FH deficient (with preserved positive internal control staining) and had classic morphology, IHC 2SC staining was deemed not necessary.

Pathological staging revealed pT3bN0M1 FH-deficient FH-RCC and multiple FH-deficient leiomyomas. Considering the multiple large uterine leiomyomas and maternal history of uterine fibroids, HLRCC is strongly indicated rather than a sporadic FH-deficient tumor. The absence of a family history of RCC could suggest variable penetrance, and, as such, it may not manifest in every carrier. Nonetheless, this reinforces the necessity of a genetic workup, which our patient is currently awaiting. Given that the waiting time for a genetics appointment is 18 months and that this tumor is known to metastasize early and rapidly progress, deferring treatment until genetic confirmation was not clinically appropriate. A pragmatic decision was made to proceed with management on the basis of clinicopathological results.

There is lack of uniformly agreed-upon, level 1 evidence for systemic therapy for metastatic FH-deficient RCC, and current guidelines recommend clinical trial enrolment (19, 20). However, clinical trial enrolment is limited and not accessible in many countries. These were challenges faced by our patient as highlighted during consultations with both the urologist and the oncologist. The most investigated regimen is bevacizumab and erlotinib. The NCCN guidelines categorize this regimen as 2A, highlighting lower-level evidence, and the ESMO Clinical Practice Guideline acknowledges that bevacizumab plus erlotinib “may be used in advanced FH-deficient RCC” but not approved by the European Medicines Agency (EMA) or the Food and Drug Administration (FDA), and is based on limited evidence (Level 3, Grade B recommendation) (20). This could have contributed to the initial delays and uncertainty in our patients’ access to systemic therapy in the national healthcare system, and reflects real-world dilemmas outside of research centers.

To date, systemic therapy trials for FH-deficient RCC have only been investigated exclusively in metastatic or advanced settings. The first is the AVATAR trial (NCT01130519), which evaluated bevacizumab (monoclonal antibody targeting BEGF-A) and erlotinib (tyrosine kinase inhibitor) (7). The AVATAR trial, consisting of 43 patients with HLRCC and 40 patients with sporadic papillary RCC, showed promising results in the HLRCC group with an overall response rate of 72%, a median progression-free survival (PFS) of 21.1 months, and an overall survival of 44.6 months. In contrast, the sporadic group showed confirmed response in 35% of patients, a median PFS of 8.9 months, and a median overall survival of 18.2 months (7). The frequently encountered treatment-related adverse events (TRAEs) in the trial were acneiform rash (91.6%), diarrhea (86.6%), and proteinuria (74.7%) with ≥Grade 3 TRAEs occurring in 52% of patients. A more recent trial evaluating the emerging combination of lenvatinib (tyrosine kinase inhibitor TKI) and tislelizumab [immune checkpoint inhibitor (ICI)] in 17 Chinese patients (NCT05877820) demonstrated an objective response rate of 93.3%, including 20% complete responses. Sixteen patients (94.1%) experienced TRAEs and four experienced ≥Grade 3 TRAEs. Notably, eight patients (47.1%) required treatment dose reduction or even discontinuation (21). This regimen highlights a challenge of treatment tolerability and requires experienced management of the side effects. Long-term survival is also yet to be observed owing to the short follow-up duration. Another trial of 41 Chinese patients (NCT04387500) evaluating sintilimab and axitinib showed an objective response rate of 56% and a median PFS of 19.8 months, with ≥Grade 3 TRAEs observed in one-third of patients, with the most common being hypertriglyceridemia (7%), rash (5%), and anemia (5%) (22). The reported median PFS of 19.8 months is comparable to the median PFS achieved with bevacizumab and erlotinib; however, overall survival data are still immature.

These studies not only illustrate an expanding treatment landscape for this tumor but also reveal limitations such as the lack of a randomized comparator arm. Our patient responded favorably to bevacizumab and erlotinib and experienced proteinuria, consistent with the known adverse event profiles. A limitation of the AVATAR trial is its largely homogeneous population; more than 80% of the enrolled patients were white and non-Hispanic. Other ethnicities, especially Chinese, as in our case, were heavily underrepresented. This case proves that the regimen can be effective even in populations not fully represented in the trial. At the time of treatment, a bevacizumab and erlotinib regimen represented the regimen with the most established evidence with the longest duration of follow-up. In contrast, the lenvatinib and tislelizumab study, while demonstrating 93.3% ORR, included 17 patients with less than a year of median follow-up duration with no reported overall survival data. Meanwhile, the trial of sintilimab and axitinib had shown an ORR of 56% and similarly lacked overall survival data. Access to tislelizumab and sintilimab was not available in Malaysia, and the trial data of lenvatinib and tislelizumab had not yet been regimen published at the time of treatment. Postoperative management in FH-deficient RCC is challenging, with no consensus on postoperative systemic therapy. This case indicates that postoperative systemic therapy may be considered in selected cases, highlighting the need for clearer guidance in this setting.

Our patient’s last imaging scan showed partial response to bevacizumab plus erlotinib, but she experienced renal impairment with proteinuria. At present, there are no standardized approaches regarding management of proteinuria observed in patients receiving bevacizumab for this subset of RCC. Owing to the patient’s single kidney post-nephrectomy experiencing worsening renal function, caution was exercised by delaying the second cycle of systemic therapy to allow for renal function recovery. The patient consulted a nephrologist who advised the discontinuation of an ACE inhibitor (Quinapril) and the initiation of an ARB (Losartan) for renal protection and sodium bicarbonate to address concurrent metabolic acidosis. This highlights the need for close renal surveillance and management including careful monitoring and early nephrology input.

**Figure 4:**
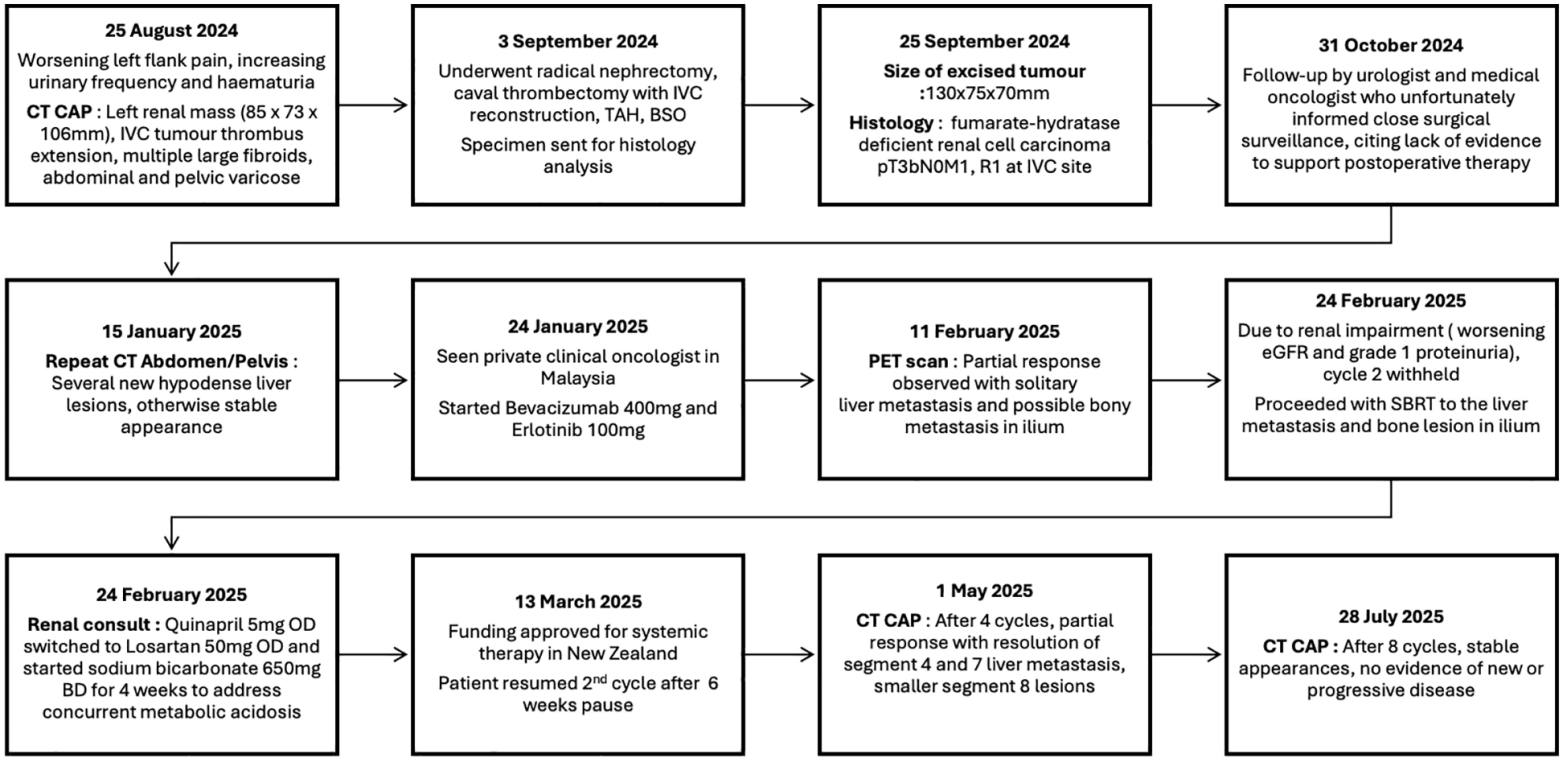
Timeline of events.

During the pause in systemic therapy, SBRT was delivered to the solitary FDG avid liver metastasis and iliac lesion as observed on PET imaging. Published case reports of metastatic FH-deficient RCC have described the use of radiotherapy predominantly in the context of bone, particularly spinal metastasis as symptom palliation or prevention of disease progression (23, 24). By contrast, our case utilized radiotherapy with a bridging intent. This decision was guided by the uncertainty of the timing of the renal function recovery and given the known aggressive nature of this particular tumor. This sequence demonstrates how SBRT can potentially achieve disease control during systemic therapy interruptions, aligning with evidence supporting the role of radiotherapy in oligometastatic RCC (20, 25). It is acknowledged that the non-irradiated segment 7 liver metastasis achieved complete response; therefore, the independent contribution of SBRT to tumor response cannot be definitively established. To the best of our knowledge, the strategy of radiotherapy delivery to liver metastasis and as a bridging-intent modality during interruption of systemic therapy has not been described in FH-deficient RCC. This reinforces the value of clinical oncology alongside medical oncology. Multidisciplinary input from joint specialties helps mitigate “professional blind spots” when established treatment guidelines are limited. The timeline of events is summarized in **Figure 4**.

## 4 Conclusion

We believe that this case will generate novel and important real-world insights into the therapeutic challenges associated with FH-RCC in settings without specialized research programs. This case highlights not only the immense burden of the disease and challenges faced by our patient but also the broader dilemmas doctors face: lack of level 1 evidence and FDA-approved therapies for this malignancy, the importance of a cautious approach to proteinuria, and addressing systemic therapy interruptions with SBRT as a possible bridging strategy. Owing to the extreme rarity of this tumor, this case contributes to the limited database of treatment responses for patients undergoing a bevacizumab and erlotinib regimen outside dedicated research centers with an expertise in this tumor type and an underrepresented patient population. Further research is needed to guide management strategies to ensure the best possible outcomes in this distinct subset of patients with RCC.

## Data Availability

The original contributions presented in the study are included in the article/supplementary material. Further inquiries can be directed to the corresponding author.
